# Clinical bacteriological aspects of the Human Amniotic Membrane in the diabetic foot. A case report

**DOI:** 10.1016/j.amsu.2020.04.020

**Published:** 2020-05-16

**Authors:** Laura Maria Chisari, Grasso Antonino, Giuseppe Chisari, Antonio Maria Borzì, Clara Grazia Chisari

**Affiliations:** a Department of Biomedical and Biotechnological Sciences, University of Catania, Italy; b Department of General Surgery and Medical Surgical Specialties, University of Catania, Italy; c Research Center “The Great Senescence”, University of Catania, Italy; d Department of Medical-Surgical Sciences and Advanced Technologies “G.F. Ingrassia”, University of Catania, Italy

**Keywords:** Skin lesions, Diabetic patient, Aerobic bacteria, Anaerobic bacteria, Skin ecosystem, Human amniotic membrane

## Abstract

The phases of a cutaneous infection are initially the adhesion of the microorganism to the cells of the host, followed by the invasion of the tissues, than the elaboration of toxins and the escape from the defense systems of the host. The Human Amniotic Membrane (HAM) is extracted from the placenta of donors after caesarean section. The amnios is characterized by a monolayer of epithelial cells, a basement membrane and an avascular stroma of collagen. The HAM showed to promote chronic wound healing. We evaluated the “in vivo” and "in vitro" activity and efficacy of the HAM in subjects with chronic diabetic skin lesion. This clinical case showed that the HAM promote the wound healing of complex chronic skin lesion in a follow-up period of 6 months after the first graft.

## Highlights

• We evaluated the Human Amniotic Membrane (HAM) activity and efficacy in the treatment of diabetic skin lesion.
• The Human Amniotic Membrane showed to facilitating cell regeneration in skin lesions.
• The Human Amniotic Membrane showed antimicrobial effects, favoring the restoration of skin surface defense systems.
• The Human Amniotic Membrane has proved to be an excellent dermal substitute for the treatment of non-healing ulcers.

## 1 Introduction

Chronic wound healing is a complex process. Numerous pathologies can affect the healing process, resulting in non-healing skin lesion and wasting of medical system resources.

Chronic wound lesion can cause devastating effect on physical and psychosocial functioning resulting in inability and depression.

Skin lesions are often a consequence of a primary or secondary microbial process due to an alteration of the host skin ecosystem. The phases of a cutaneous infection are initially the adhesion of the microorganism to the cells of the host, followed by the invasion of the tissues, the elaboration of toxins and the escape from the defense systems of the host itself. The Human Amniotic Membrane (HAM) is extracted from the placenta of donors after caesarean section. The criteria for HAM extraction are provided by the National Guidelines of the National Transplantation Center [[1], [2], [3], [4], [5]]. The HAM has a therapeutic potential due to the properties of its components. The HAM is rich in extracellular matrix with a healing action for the vascular tissues. Experimental studies "in vivo" have highlighted the importance of its action in the re-epithelization of the ocular surface [[6], [7], [8], [9]]. Other studies showed the safety, ease of use and considerable benefit in facilitating cell regeneration in skin lesions. Davis in 1910 first used the HAM as a skin graft; then Sabella in 1913, followed by Douglas in 1952, used the HAM as a permanent cover on injuries of burn patients. Factors that impeded the development of HAM use in the past, were the lack of techniques of conservation and infectious screening. The HAM develops from an extra-embryonic tissue and includes two fetal layers: the chorion (external) and the amnios (internal). The extracellular membrane is made up of three primary biomolecules: specialized proteins (Fibronectin, TIMP and Laminine); proteoglycans and structural proteins (Collagen type I, III, IV, V, VI and elastin). The HAM showed different properties: high cell regeneration stimuli due to the expression of growth factors (EGF, TGF, FGF, PDGF A and B); poor immunogenicity, due to the lack of expression of HLA A, B, C, or beta-2 microglobulin antigens and anti-inflammatory and regulating healing effect, reduction of TGF-β receptor expression on fibroblast metalloproteases and suppression of pro-inflammatory cytokines (IL-1RA and IL-10) [[11], [12], [13], [14], [15]]. The HAM showed antimicrobial effect similar to innate immunity, such as β-defensins, elastase inhibitors and leukocyte proteinase inhibitors, lactoferrin and IL-1RA. Both lactoferrin and IL-1RA showed a high antimicrobial and anti-inflammatory effect. We evaluated the “in vivo” and "in vitro" activity and efficacy of the HAM in subjects with diabetic foot. The aim of this case report was to demonstrate the efficacy of the HAM in cell regeneration in a follow-up period of 6 months from graft.

## 2 Methods

The HAM was applied to the wound after surgical toilet when the lesion stop healing. Surgical toilets of the lesion were performed 5 days after the HAM application, than every 15 days. Dressing were covered with inert material (Mepitel type), than sterile gauze and bandage. All procedures were performed by a trained consultant angiologist.

This procedure was registered at www.researchregistry.com as “researchregistry5345”.

Bacteriological tests for aerobic and opportunistic anaerobic bacteria of the wound were performed before every HAM application.

This case report was described according to the SCARE guidelines [10].

## 3 Case presentation

We report the case of a 69 years old male diabetic patient with chronic ulcers treated with HAM. The patient attended to our clinic in November 2013. Patient's account of the medical history: smoking habits, arterial hypertension, chronic peripheral obliterative arteriopathy stage IV, chronic ulcers in the left lower limb. The patient was under medical treatment for diabetes mellitus and hypertension. The patient had no other medical or surgery history, and had no relevant genetic, family or psychosocial history. Bacteriological tests for aerobic and opportunistic anaerobic bacteria were negative. The patient underwent wound medications up to 2014, then he was treated with angioplasty with drug-eluting balloons of left popliteal artery and superficial femoral artery, femoral-tibial by-pass, amputation of the first toe of left foot and graft of dermal substitute (INTEGRA). After ten months, a medullary electrostimulator was placed for pain control, following the amputation of left foot. The bacteriological tests performed on the wound were negative. The amputation was followed by four surgical toilets of the stump with HAM grafting every five months. The hyperbaric oxygen therapy was not performed on the patient because the chronic wound was a nonrevascularizable IV stage Leriche-Fontain lesion. Surgical toilets were performed every 15 days. The complete healing of the lesion was reviewed after twenty months. The patient correctly follows all suggestion for wound dressing and medical appointment.

## 4 First graft

First graft with HAM, measuring 10 × 12.5 cm;

**Figure:**
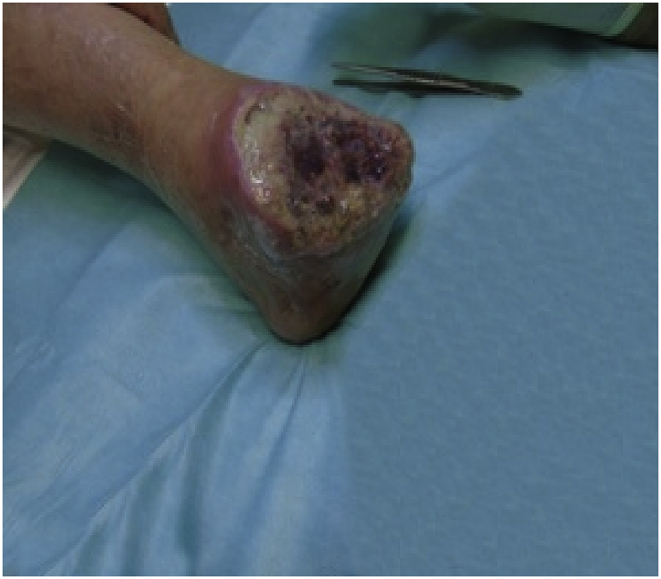

We applied inert material (Mepitel type) and covered with sterile gauze and bandage.

**Figure:**
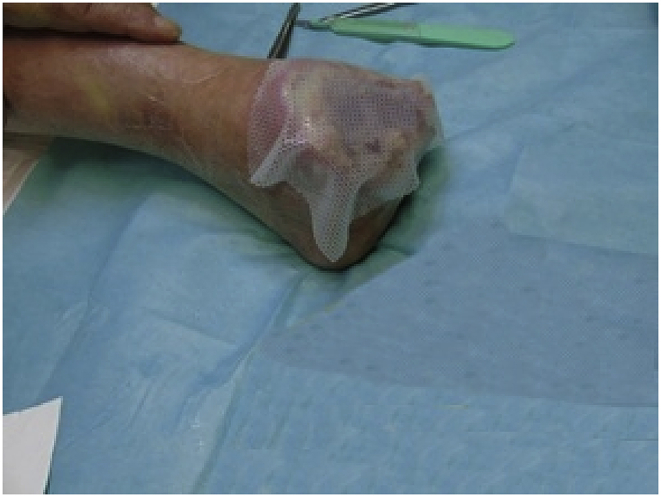

First post application treatment after five days. The patient reported significant pain relief and had returned to walking.

## 4.1 Second graft

After a period of well-being, which lasted about a year, the lesion stop healing, we grafted a second HAM (10 × 12.5 cm).

**Figure:**
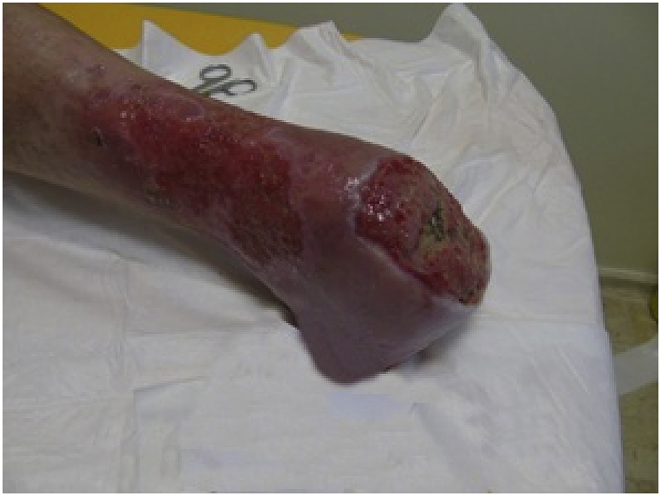

## 4.2 Third graft

Given the excellent results obtained and the small residual area of the lesion, we decided to graft a new smaller HAM (6 × 6 cm).

**Figure:**
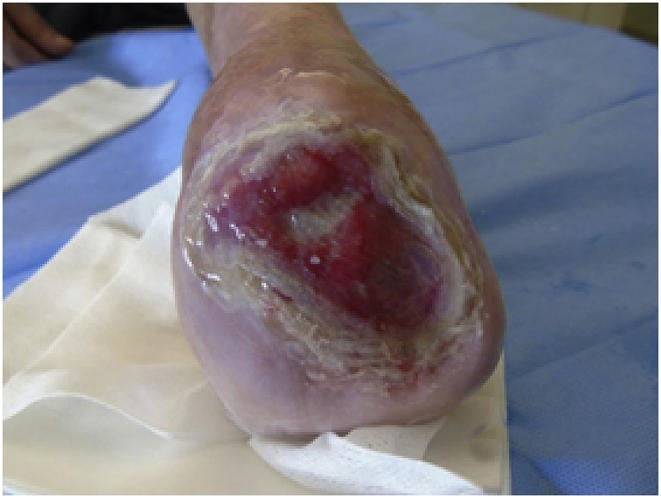

## 4.3 Fourth graft

Given the small size of the lesion, we choose to graft the last HAM (3 × 3 cm).

**Figure:**
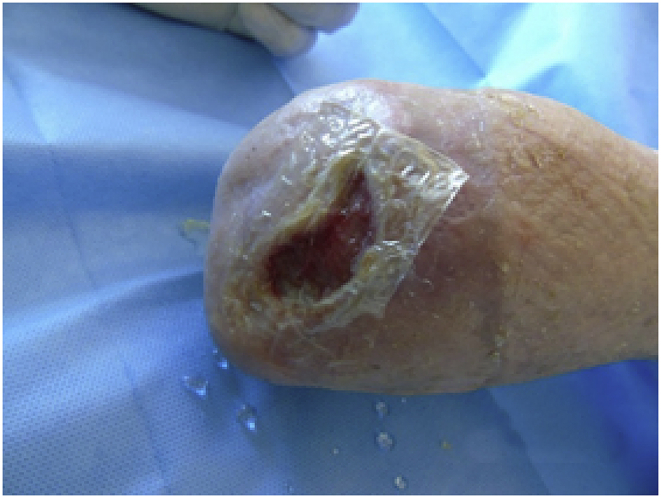

## 4.4 Healing results

The pain disappeared when the lesion healed completely. No infection was detected during the follow-up.

**Figure:**
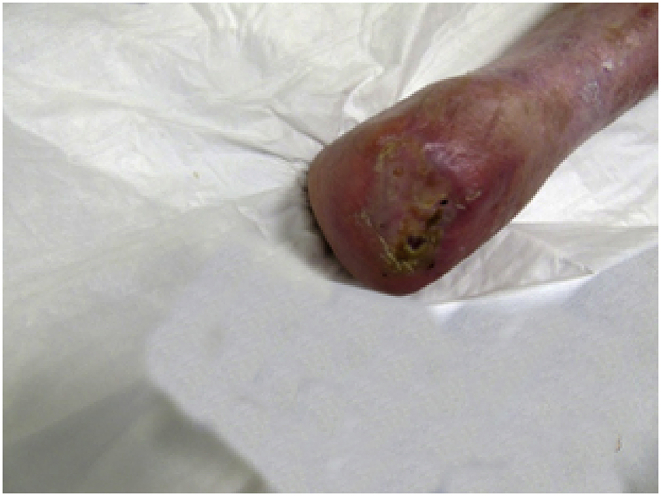

After complete healing, the skin swab performed on the healing surface showed the presence of normal Gram positive bacteria of the skin such as Staphylococcus epidermidis.

**Figure:**
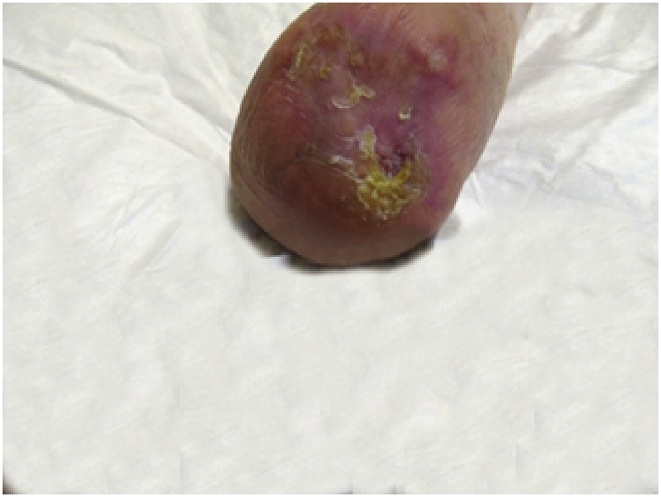

## 5 Conclusions

The normal skin microbiota represents one of the main skin defense barrier. The etiology of skin infections in diabetic patients is mainly polymicrobial, with a predominant role of Gram-positive pathogens such as *Staphylococcus aureus*, Staphylococcus epidermidis, Streptococcus piogene, Corynebacterium spp, and some Gram-negative bacilli (aerobic and anaerobic) [[1], [2], [3], [4], [5], [6], [7], [8], [9]]. Non-infectious chronic skin lesions from decubitus ulcers and diabetic foot may be the site of secondary bacterial infections. The identification of pathogens responsible for secondary bacterial infections plays a crucial role in choosing the best treatment. Recent studies showed an emerging antibiotic resistance of these bacterial pathogens in secondary infections. In our case, we found negative bacteriological test results during the healing process [[16], [17], [18], [19], [20]]. After complete healing of the lesion of the foot, a presence of Staphylococcus epidermidis was found to confirm the restoration of the normal skin microbiota. This case confirmed that the HAM, in addition to surgical toilets, favor wound healing processes, also intervenes in the restoration of skin surface defense systems and has proved to be an excellent dermal substitute for the treatment of non-healing ulcers.

Finally, the HAM promote the healing of chronic wound, exerts antimicrobial effects and reduces significantly pain leading to an improvement of the quality of life. However, clinical trial are needed to better investigate the role of HAM in the treatment of chronic wound.

## Ethical approval

This research was conducted in accordance to the ethical standard.

## Sources of funding

This paper was not funded.

## Author contribution

Laura Maria Chisari: study concept and writing the paper.

Grasso Antonino: data collection and study concept.

Giuseppe Chisari: data analysis and interpretation, writing the paper.

Antonio Maria Borzì: data analysis or interpretation, writing the paper.

Clara Grazia Chisari: data analysis or interpretation, revision.

## Guarantor

Professor Antonino Grasso

## Provenance and peer review

Not commissioned externally peer reviewed.

## Declaration of competing interest

The Authors declare no conflicts of interest.
